# Transcriptomic Analysis of Grapevine (cv. Summer Black) Leaf, Using the Illumina Platform

**DOI:** 10.1371/journal.pone.0147369

**Published:** 2016-01-29

**Authors:** Tariq Pervaiz, Jia Haifeng, Muhammad Salman Haider, Zhang Cheng, Mengjie Cui, Mengqi Wang, Liwen Cui, Xicheng Wang, Jinggui Fang

**Affiliations:** 1 Key Laboratory of Genetics and Fruit development, College of Horticulture, Nanjing Agricultural University, Nanjing, 210095, P. R. China; 2 Jiangsu Academy of Agricultural Sciences, Nanjing, P. R. China; Hainan University, CHINA

## Abstract

Proceeding to illumina sequencing, determining RNA integrity numbers for poly RNA were separated from each of the four developmental stages of cv. Summer Black leaves by using Illumina HiSeq^™^ 2000. The sums of 272,941,656 reads were generated from *vitis vinifera* leaf at four different developmental stages, with more than 27 billion nucleotides of the sequence data. At each growth stage, RNA samples were indexed through unique nucleic acid identifiers and sequenced. KEGG annotation results depicted that the highest number of transcripts in 2,963 (2Avs4A) followed by 1Avs4A (2,920), and 3Avs4A (2,294) out of 15,614 (71%) transcripts were recorded. In comparison, a total of 1,532 transcripts were annotated in GOs, including Cellular component, with the highest number in “Cell part” 251 out of 353 transcripts (71.1%), followed by intracellular organelle 163 out of 353 transcripts (46.2%), while in molecular function and metabolic process 375 out of 525 (71.4%) transcripts, multicellular organism process 40 out of 525 (7.6%) transcripts in biological process were most common in 1Avs2A. While in case of 1Avs3A, cell part 476 out of 662 transcripts (71.9%), and membrane-bounded organelle 263 out of 662 transcripts (39.7%) were recorded in Cellular component. In the grapevine transcriptome, during the initial stages of leaf development 1Avs2A showed single transcript was down-regulated and none of them were up-regulated. While in comparison of 1A to 3A showed one up-regulated (photosystem II reaction center protein C) and one down regulated (conserved gene of unknown function) transcripts, during the hormone regulating pathway namely SAUR-like auxin-responsive protein family having 2 up-regulated and 7 down-regulated transcripts, phytochrome-associated protein showed 1 up-regulated and 9 down-regulated transcripts, whereas genes associated with the Leucine-rich repeat protein kinase family protein showed 7 up-regulated and 1 down-regulated transcript, meanwhile Auxin Resistant 2 has single up-regulated transcript in second developmental stage, although 3 were down-regulated at lateral growth stages (3A and 4A). In the present study, 489 secondary metabolic pathways related genes were identified during leaf growth, which mainly includes alkaloid (40), anthocyanins (21), Diterpenoid (144), Monoterpenoid (90) and Flavonoids (93). Quantitative real-time PCR was applied to validate 10 differentially expressed transcripts patterns from flower, leaf and fruit metabolic pathways at different growth stages.

## Background

*Vitaceae* comprised of 14 genera and about 900 species which are distributed all over the globe particularly in tropical regions of Australia, Asia, Africa and the Pacific Islands with a small number of genera in temperate areas [1]. *Vitis* is one of the economically most important and globally cultivated fruit crop, covering about 8 million hectares of area and producing about 67.5 million tons (http://www.oiv.int/) of grape berries, with highly valued products such as juices, liquors and wines [2].

Leaf is the most important vegetative organ supplying the energy, nutrition and hormones for the fruit growth and development. Thorough knowledge of the ongoing biological networks during the leaf growth is very important. So leaf growth and development in all vascular plants is begun with the growth of meristem tissues. In plants, subsequent cell multiplication and elongation occurs primarily from a basal intercalary meristem producing a gradient of cells along the leaf, with the sequence of interconnected and overlapping phases: initiation, general cell division, transition, cell expansion and meristemoid division phases [3]. Consecutive transverse segments of leaf therefore provide harmonized large number of cells at various developmental stages. Successive transverse sections of a leaf provides synchronized number of cells at different growth stages [4]. The hypotheses of stage specific genes are more important for growth is also support the findings of Fasoil et al., [5], he reported that the organ identity in the grapevine transcriptome is less important than the developmental stage. Few organ-specific genes were shared among the different developmental stages; however up to 16% of the organ-specific genes expressed in the flower were common to the different floral organs.

Photosynthetic differentiation is essential for vascular plants; however it is not clearly understood. Although various pathways related to leaf development have been thoroughly reviewed, to understand the viewpoint of the single cell has not been discovered scientifically [6]. The studies were focused mainly on leaf development and networks of genes signaling, that are linked with the conscription of cells from meristem develops into leaf primordia, the organization of abaxial-adaxial polarity and the expansion of the blade laminal [7]. As a result, an incomplete thoughtful knowledge about signaling coordination that compels photosynthetic progress from proplastid to chloroplast [8]. Leaf development is concerned with the photosynthetic productivity which underlies the global challenges such as climate change, bio-energy and ensure food security; provides thoughtful knowledge and enables us to operate photosynthetic activities [3]. Additional research work predicted the preliminary knowledge about the mechanisms that direct the trafficking of chlorophyll metabolic intermediates in leaves. In plants photosynthetic activities are more complex due to chloroplastic and cellular dimorphism. photosynthesis relies on Kranz-type leaf anatomy in which the veins running the size of the leaf are enclosed by two layers [9]. While much progress has been made in defining growth regulators signaling and biosynthetic pathways, but the regulation by ecological and growth signals remain not well reported. *De novo* auxin biosynthesis plays an indispensable role in plant growth. The growth hormone auxin, which is mainly symbolized by indole-3-acetic acid, is concerned with the regulation of plant development. Even though IAA was the primary plant hormone acknowledged, the biosynthetic pathway at the genetic level has remain unclear [10].

Transcriptome sequencing using NGS technologies have been increasingly carried out in model as well as in non-model plants for gene detection and advancement in markers development [11, 12]. Due to the rapidly developing technology of NGS, the quantity of sequencing data that could be produced in experiment has radically increased in current years, as the total length of the sequencing reads. This has led to a better level of transcriptome exposure, enhanced the specificity and precision as in mapping sequencing reads. Significantly, constant incremental developments in defining the grapevine transcriptome in the form of functional annotation [13, 14] and gene ontology assignment [15], now permits the precise narration of the functional task of mainly about *Vitis vinifera* genes [16]. Particularly analogous RNA deep-sequencing presents modern scientific platform for scrutinizing transcriptional instruction. It makes possible the accurate elucidation of transcripts present surrounded by a specific sample, and could be applicable to work out gene function based on unconditional transcript abundance [17]. In the single acknowledgment, in grape vine Zenoni, Ferrarini [18] produced RNA sequencing information from *Vitis vinifera*, and presented the preliminary indication about the multifarious process of gene expression and regulation throughout grape berry growth.

The present study was designed to find out the transcriptional system that is connected with the development of leaves and production of metabolites during photosynthesis. We have examined the cellular expression by using up-to-date RNA sequencing expertise to conduct a complete analysis of the transcriptional profile of *V*. *vinifera* leaves at four different developmental stages. We studied the reference transcriptomes for RNA-Seq analysis, validate several transcriptional changes recorded with the help of quantitative real-time PCR and validated with the flower fruit and leaf growth stages, metabolic pathways described the biological processes that are supplemented in differentially regulated transcripts. We explored these data with Bioinformatics tools to identify the reprogramming of major metabolic activities along the leaf gradient.

## Material and Methods

### Leaf Sample collection

**Fig 1:**
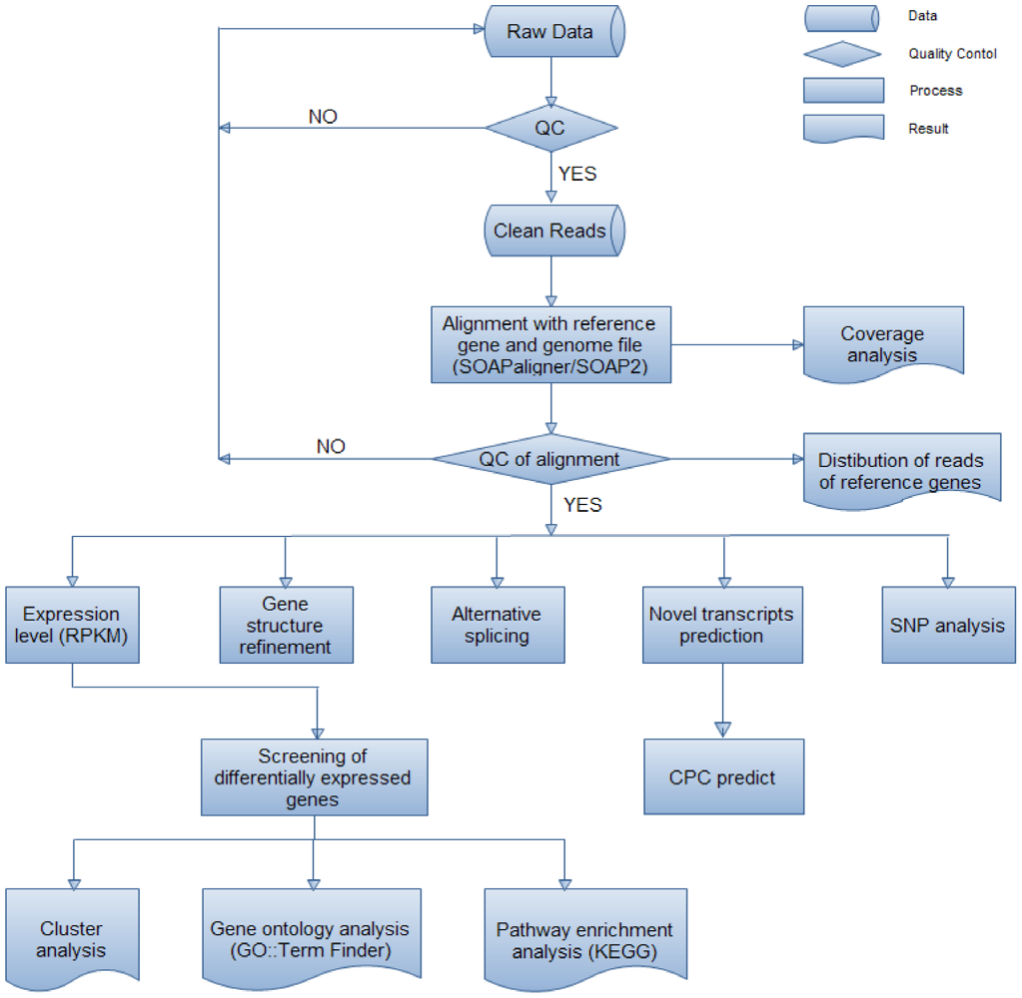
Four representative samples of grapevine leaf (1A, 2A.3A and 4A).

Cv. Summer Black (hybrid of *V*. *vinifera* and *V*. *labrusca*) trees were grown under the standard cultivation conditions at the Jiangsu Agricultural Research Station Nanjing, China. We selected 5–6 years old plants for sampling during spring to summer growing season, four representative growth stages according to cell multiplication and expansion defined stages (Fig 1), from the start to the rapid expansion phase, young leaves (2 weeks old 1A), medium size leaves (5 weeks old 2A), large leaves (7 weeks old 3A) and mature leaves (10 week old 4A) with the three replication for each growth stage were collected and immediately frozen in liquid nitrogen, and subsequently stored at -80°C for further use.

### RNA extraction, cDNA library construction and Illumina deep sequencing and Analysis of gene expression level

Total RNA samples from selected samples with three replication of each sample were extracted by using Trizol reagent (Invitrogen, Carlsbad, CA, USA) and subsequently used for mRNA purification and library construction with the Ultra^™^ RNA Library Prep Kit for Illumina (NEB, USA) following the manufacturer’s instructions. The samples were sequenced on an Illumina Hiseq^™^2000. Sequencing was completed by the Shanghai Hanyu Biotechnology Company (Shanghai, China). Primary sequencing data that produced by Illumina HiSeqTM 2000, raw reads, is subjected to quality control (QC) that determine if a resequencing step is needed. After QC, raw reads are filtered into clean reads and were aligned to the *vitis vinifera* reference mRNA and genome http://www.genoscope.cns.fr/externe/GenomeBrowser/Vitis/ using SOAP2 http://soap.genomics.org.cn/soapaligner.html, version 2.21 with default settings. QC of alignment is performed to determine if resequencing is needed. After alignment result passed QC, we precede analysis, these findings of gene expression including, differential expression analysis and gene expression levels. Further, we performed Pathway enrichment analysis and Gene Ontology (GO) enrichment analysis. SAM (shoot apical meristem) same tools and BamIndexStats.jar were used to calculate the gene expression levels, and RPKM value from SAM files. Gene expression difference between log and early stationary phase were obtained by MARS (MA-plot-based method with Random Sampling model), a package from DEGseq [52]. We simply defined genes with at least 1-fold change between two samples and FDR (false discovery rate) less than 0.001 as differential expressed genes. Transcripts with |log2FC| < 1 were assumed have no change in expression levels.

### Pathway enrichment analysis of DEGs

Genes usually interact with each other to play important role in certain biological functions. Pathway-based analysis helps us to further understand genes biological functions. KEGG (the main publicly available database related to pathway) is used to perform pathway enrichment analysis of DEGs [22]. This analysis identifies significantly enriched signal transduction pathways or metabolic networks in DEGs, evaluate with the whole genome background. The calculating principle is the same as that in Gene Ontology analysis. Here M is the number of all genes annotated to specific pathways, N is the number of all genes that with KEGG annotation, n is the number of DEGs in N, m is the number of DEGs in M.

$$P=1-\overset{m-1}{\underset{i=0}{\sum }}\frac{\left(Mi\right)\left(\begin{array}{c} N-M\\ n-i \end{array}\right)}{\begin{array}{c} N\\ n \end{array}}$$

### qRT-PCR Validation

qRT-PCR was performed to verify the expression patterns revealed by the RNA-seq study. We used samples at the developmental stages of flower (Fl), 4 stages of fruit (Fr) and 4 stages of leaf (Le). The purified RNA samples were reverse-transcribed using the PrimeScript RT Reagent Kit with gDNA Eraser (Takara, Dalian, China) following the manufacturer’s protocol. Ten transcripts were selected randomly for the qRT—PCR assay. Gene specific qRT—PCR primers were designed using Primer3 software (http://primer3.ut.ee/), for 10 selected genes with the sequence data in the 3’ UTR “S1 File”. qRT-PCR was carried out using an ABI PRISM 7500 real-time PCR system (Applied Biosystems, USA). Each reaction contains 10μl 2×SYBR Green Master Mix Reagent (Applied Biosystems, USA), 2.0μl cDNA sample, and 400 nM of gene-specific primer in a final volume of 20μl. PCR operating conditions were at 95°C for 2 min, followed by 40 cycles of heating for 10s at 95°C and annealing temperature at 60°C for 40 s. The relative mRNA expression level for apiece gene was computed as ΔΔCT values [53]. A primer pair was also designed for TC81781 (Release, 6.0 Institute for Genomic Research), encoding an actin protein. qRT-PCR analysis were performed with three replicates for each cDNA sample.

## Results and Discussion

### Sequence quality control and Illumina HiSeq RNA sequencing

**Fig 2:**
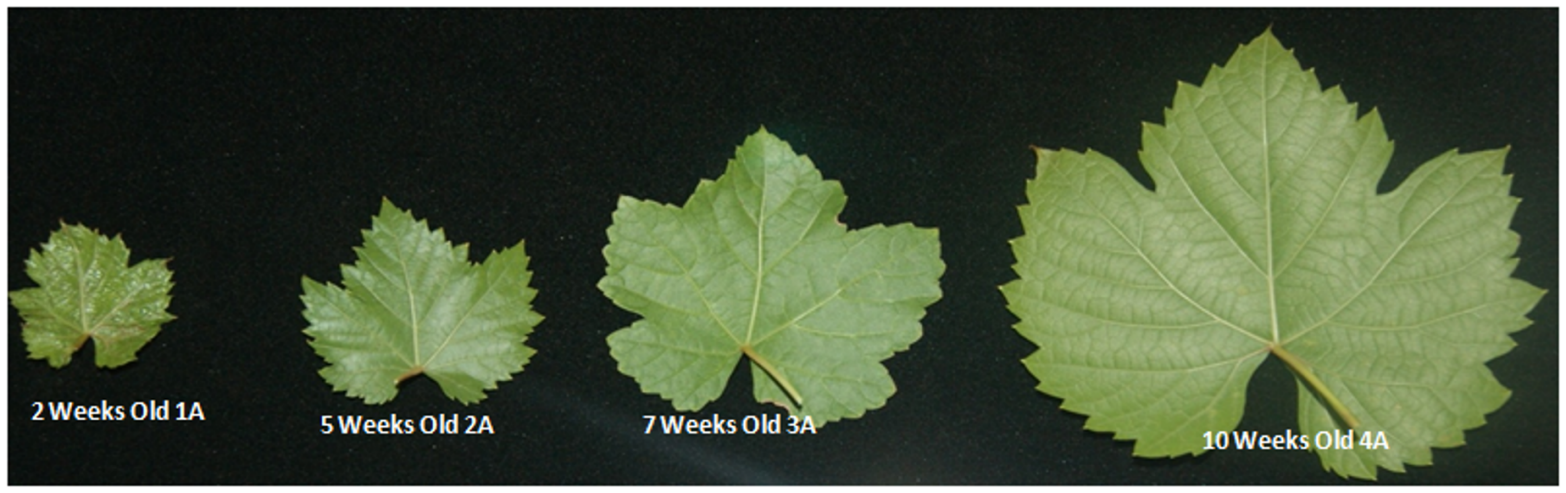
Overall strategy of leaf Illumina Transcriptome Sequencing, data analysis and Bioinformatics analysis pipeline.

Primary sequencing data that produced by Illumina HiSeq^™^ 2000, raw reads were subjected to quality control (QC) that determined, if may be re-sequencing step is needed. After QC, raw reads were filtered into clean reads which were further aligned into the reference sequences with SOAPaligner/SOAP2 [19]. The alignment data was utilized to compute reads distribution on grape reference genome and performed coverage analysis. Alignment result passed QC, and then proceeds with further analysis, which includes gene expression, gene structure refinement, alternative splicing, novel transcript prediction and annotation and SNP detection. Results of gene expression include differential expression analysis and gene expression levels. In addition, we performed Gene Ontology (GO) enrichment analysis and Pathway enrichment analysis. The detailed stratagem is schematically presented in Fig 2. The *de novo* assembly quality dependents on the choice of an assembler pursued by parameters coverage, N50 Value, and Hash length. The quality of the transcriptome assembly might be determined by the assembly software that is employed to assemble the short read data [20]. Finally, sums of 272941656 reads were generated from all biological replicates. Proceeding to illumina sequencing, determining RNA integrity numbers for poly RNA were separated from each of the four developmental stages of leaves using Illumina HiSeq^™^ 2000. RNA sample from each stages was sequenced on a single lane of an Illumina HiSeq 2000 and indexed with unique nucleic acid identifiers. Total 272,941,656 reads were produced, with the total sequence data of more than 27 billion nucleotides. This match up to favorably among the 2.2 billion nucleotides data comprising of 59 million 36–44 bp reads generated during the earlier studies on grapes transcriptome sequencing investigation project [18]. Furthermore longer and higher sequence coverage read length enables much greater precision with the mapping to reference transcriptomes or genomes. De-multiplexing with the unique identifiers revealed the data consist of 69,651,180 reads from leaf sample 1A, 2A (63844540), 3A (64101070) and 4A (75344866), respectively (Table 1). These findings suggests that the data is almost 10-fold higher than reported by [21] in Chinese bayberry during fruit development through Illusmina-based transcriptome analysis (5.3 million 90 bp reads).

**Table 1:** Number of reads sequenced and mapped to the grapevine genome.

	1Ayoung leaves(2 weeks)	2Amedium size leaves (5weeks)	3Alarge leaves(7 weeks)	4Amature leaves (10 week)	Sum
**Clean Reads**	69651180	63844540	64101070	75344866	272941656
**Total mapped**(percent of clean reads)	5445963478.19%	4935637177.31%	5010660378.17%	5851461177.66%	212437219
**Unique match**(percent of clean reads)	5420473877.82%	4910860876.92%	4985461277.78%	5822933177.28%	211397289
**Mutliple match**(percent of clean reads)	254896 0.37%	247763 0.39%	251991 0.39%	285280 0.38%	1039930
**Total unmapped**(percent of clean reads)	15191546 21.81%	14488169 22.69%	1399446721.83%	1683025522.34%	60504437

### Global transcriptome analysis, functional classification, and metabolic pathway analysis by KEGG of all detected transcripts

To monitor the leaf growth and development, the growth stages of leaf such as young leaves (2 weeks old), medium size leaves (5 weeks old) large leaves (7 weeks old), and mature leaves (10 week old)) were carefully selected and harvested. The RNA of grapevine leaf was used for deep sequencing through Illumina HiSeq 2000 platform. After selecting the raw reads from all four developmental stages of leaf, 1A, 2A, 3A and 4A revealed 69.6, 63.8, 64.1 and 75.3 million clean reads, respectively, corresponding to 21.22 Gb clean data were received. The sequence reads were aligned to the grapevine reference genome using SOA Paligner/soap2 software (http://soap.genomics.org.cn/), allowing at least two base mismatches. From the total reads, Total mapped Unique matches Mutliple matches and Total unmapped sequences showed, 77.66%, 77.28%, 0.38% and 22.34% genomic locations, respectively (Table 1).

Genes usually interact with each other to play roles in certain biological functions. Pathway-based analyses help us to further understand genes biological functions. Kyoto Encyclopedia of Genes and Genomes (KEGG) [22] is used to perform pathway enrichment analysis of DEGs (the major common pathway-related catalog). This investigation classifies signal transduction pathways or significantly enriched metabolic networks in DEGs matched with the entire genome. The Pathway database KEGG, records the networks of molecular interactions in the cells and variants of them specific to particular organisms. Detecting the most significant pathways, the enrichment analysis of DEG pathway significance, allow us to see detailed pathway information in KEGG database. Pathway-based analyses facilitate to understand the biological functions and interactions of genes [20]. To further investigate the transcripts of *vitis vinifera*, KEGG pathway database was used to analyze transcripts. KEGG annotation results were retrieved from KEGG database based on sequence similarity, 1Avs2A 770, 1Avs3A 1385, and 3Avs4A 2294 out of 15614 (71%) transcripts were recorded in KEGG pathways. Differentially expressed genes in leaf during four stages were found in this study (S3 File), which appeared to be mainly involved in “Biosynthesis of secondary metabolites” (539 transcripts), “Metabolic pathways” (771 transcripts), “Starch and sucrose metabolism” (82 transcripts) and “Plant hormone signal transduction” (248 transcripts) were involved.

**Fig 3:**
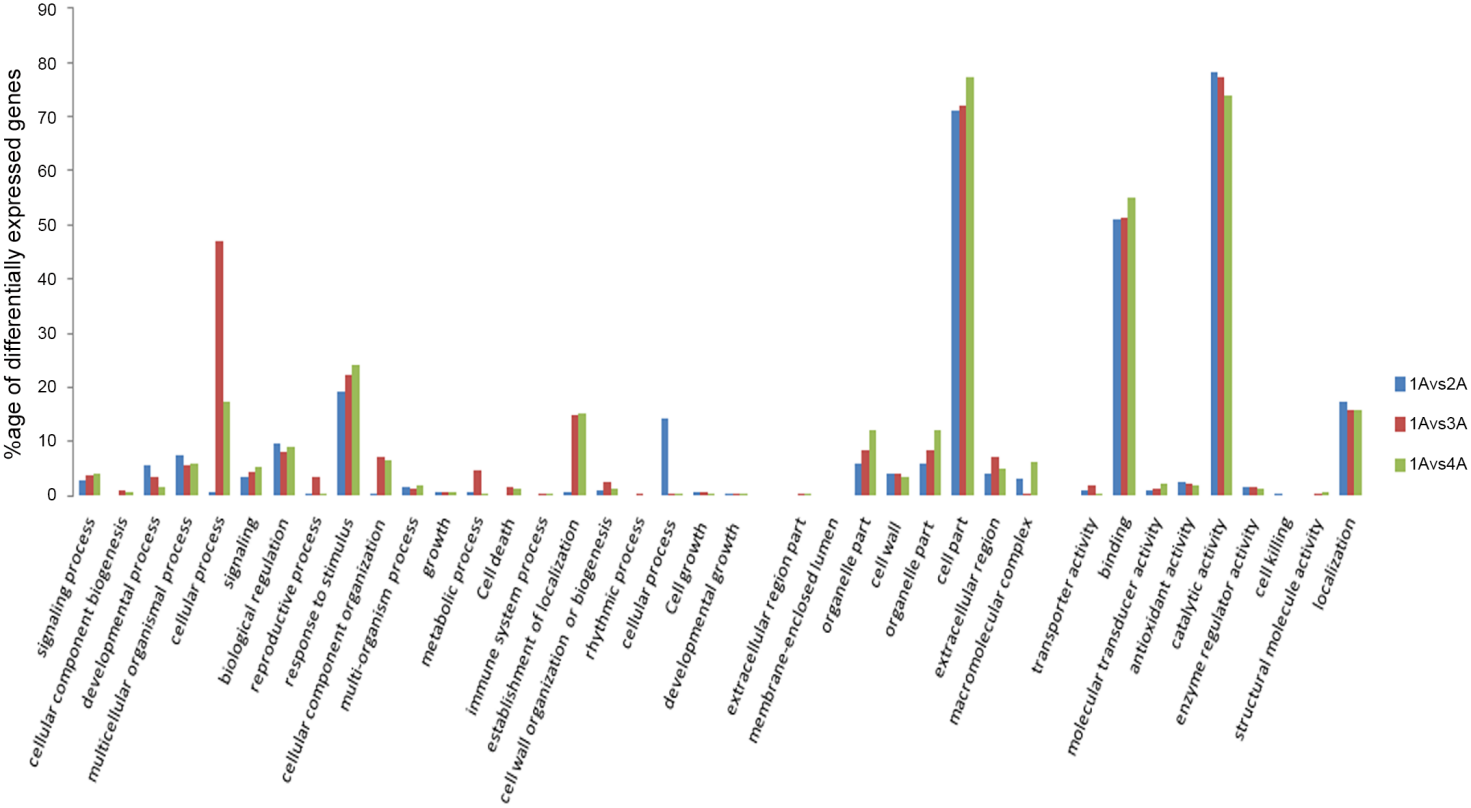
Differentially expressed genes in Molecular function, Cellular component and Biological process.

**Fig 4:**
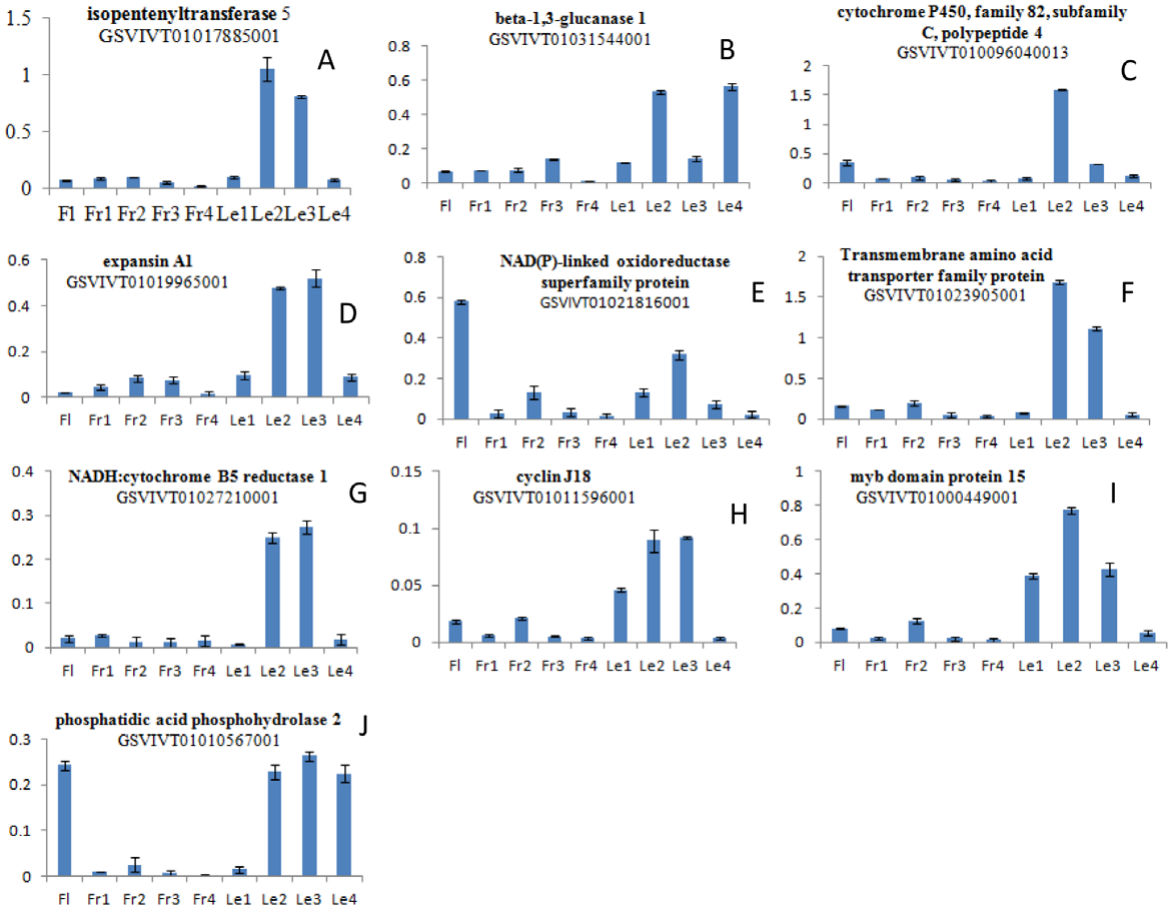
Quantitative RT-PCR validation of differential expressed transcripts observed for 10 selected genes, measured by RNA-Seq and qRT-PCR. (Fl; Flower, Fr; Fruit, Le; Leaf).

In order to determine the differentially expressed genes in Molecular function, Cellular component and Biological process among the developmental stages of leaf, gene ontology (GO) based enrichment tests were performed (Fig 3). In the comparison of GOs within and among leaf samples at specific developmental stages, the following transcripts were recorded. In the comparison of 1Avs2A, a total of 1532 transcripts were annotated in GO, including Cellular component, “Cell part” 251 out of 353 transcripts (71.1%), intracellular organelle 163 out of 353 transcripts (46.2%) and transferase activity 147 out of 654 transcripts (22.5%) in molecular function, and metabolic process 375 out of 525 (71.4%) transcripts, multicellular organism process 40 out of 525 (7.6%) transcripts in Biological process were most common. While in case of 1Avs3A, cell part 476 out of 662 transcripts (71.9%), and membrane-bounded organelle 263 out of 662 transcripts (39.7%) were recorded in Cellular component. Catalytic activity 914 out of 1183 transcripts (77.3%), transferase activity 304 out of 1183 transcripts (25.7%) in molecular function, while single-organism process 240 out of 953 transcripts (25.2%), localization 151 out of 953 transcripts (15.8%) in biological processes were predicted. The comparison of 1A with 4A, membrane with the highest transcript number having 453 out of 1439 transcripts (31.5%,) in Cellular component, while in Molecular function catalytic activity 1737 out of 2350 transcripts (73.9%) and single-organism process 479 out of 1851 transcripts (25.9%) in Biological process were annotated (Fig 3), response to stress 195 out of 1931 transcripts (10.1%) in Biological process (Table 2). Although 3Avs4A intracellular 603 out of 1029 transcripts (58.6%), organelle 538 out of 1029 transcripts (52.3%) in Cellular component, catalytic activity 1324 out of 1772 transcripts (74.7%) in Molecular function and cellular process 718 out of 1406 transcripts (51.1%), organic substance metabolic process 570 out of 1406 transcripts (40.5%) biological process were involved (**S 2**). We validated the observed transcripts by using qRT-PCR of differential expressed genes at specific growth phases of flower, fruit and leaf (Fig 4), results showed that the expression pattern between the all four growth phases were consistent across all biological samples, and extremely stage specific expression differences observed among all developmental phases for molecular function, cellular component and biological process were consistent in across three biological replicates as also mentioned by Sweetman, Wong (16].

**Table 2:** Gene ontology (GO) Molecular function, Cellular component and Biological process among the leaf developmental stages.

	1Avs2A	1Avs3A	1Avs4A	2Avs3A	2Avs4A	3Avs4A	Total
**Cellular component**	353	662	1439	415	1477	1029	5375
**Molecular function**	654	1183	2350	697	2381	1772	9037
**Biological process**	525	953	1851	582	1931	1406	7248
	1532	2789	5640	1694	5789	4207	21660

### Identification of expressed genes responding to leaf developmental stages

One of the major goals of transcriptome sequencing is to compare gene expression levels between leaf samples and within developmental stages. In 1Avs2A total, 22,153 transcripts expressed in the grapevine leaf transcriptome, including 18727 transcripts showed no significant expressional changes (|log2 fold-change (log2FC)|<1) during the first developmental stage, 2032 showed significantly up-regulated and 1394 were down-regulated. While 1Avs3A total 21973 transcripts expressed in the grapevine leaf, including 17414 transcripts showed no significant expressional changes (|log2 fold-change (log2FC)|<1), with 1566 showed significantly up-regulated and 2993 were down-regulated. Whereas 1Avs4A, a total of 22290 transcripts expressed, including 15048 transcripts showed no significant expressional changes (|log2 fold-change (log2FC)|<1), with 2305 showed significantly up-regulated and 4963 were down-regulated. Although in case of second developmental stage 2Avs3A, total 22078 transcripts were expressed in the grapevine leaf transcriptome, including 1130 transcripts showed no significant expressional changes (|log2 fold-change (log2FC)| < 1), 755 showed significantly up-regulated and 2815 transcripts were down-regulated. During the final stages of leaf development 3Avs4A, total 22069 transcripts were expressed in the grapevine leaf, including 14954 transcripts showed no significant expressional changes (|log2 fold-change (log2FC)| < 1), 2384 showed significantly up-regulated and 2737 were down-regulated.

### Leaf growth and development alter photosynthesis and chlorophyll metabolic pathway in grapevine

Chlorophyll plays significant role and acts as bio-molecule in photosynthesis, which allows plants leaves to absorb energy from sun light to convert solar energy into chemical energy. Leaf growth has significant effect on chlorophyll (chl) contents and photosynthetic functions in leaves. In this study, we observed leaf development and the levels of different genes expression in comparison with the leaf initiation to maturity stages, which have showed marked changes in photosynthetic and chlorophyll content at particular developmental stages, it also exhibits the specific roll of genes and their nature of expression.

The chlorophyll synthetic pathway generally comprised on (I) chlorophyll A synthesis from glutamate, (II) inter-conversion of chlorophyll A and chlorophyll B (chlorophyll cycle), and (III) the chlorophyll degradation pathway. In grapevine transcriptome, during the initial stages of leaf development in comparison with 1Avs2A only single transcript (photosystem I reaction center subunit PSI-N, chloroplast, putative / PSI-N, putative (PSAN)) showed down-regulated and none of them were upregulation was observed during this stage (Table 3). While the comparison of 1A to 3A showed the one up-regulated (GSVIVT01006662001, photosystem II reaction center protein C) and one down regulated (GSVIVT01022171001, Conserved gene of unknown function) transcripts, although in comparison between 1A and 4A showed 5 transcripts including 4 up-regulated and single transcript was down-regulated (photosynthetic electron transfer C PetC). However comparing the stage 2A with 3A showed significantly increased number of transcripts, 10 showed significantly up-regulation(photosystem II reaction center protein D PsbD, photosystem II subunit O-2 PsbO, Photosystem II reaction center PsbP family protein, Photosystem II reaction center PsbP family protein, photosystem II reaction center protein D, photosystem I subunit D-2, and three were down-regulated (E2F-related protein PetB, photosynthetic electron transfer C PetC, GSVIVT01000982001, PetF). In comparison of 3A with 4A, 5 transcripts were up-regulated (photosystem II reaction center protein B PsbB, photosystem II reaction center protein A PsbA, electron carriers, PsbE, GSVIVT01001159001 PsaD, Cytochrome C PetJ) and one is down-regulated (photosynthetic electron transfer C PetC) (Table 3).

**Table 3:** Differential expressed genes, related to Porphyrin and chlorophyll metabolism.

Gene ID	Trait	Log2FC	FDR	Leaf stage	Result
GSVIVT01030293001	ferritin 4	3.86844	1.65E-30	1Avs2A	Up
GSVIVT01019612001	NAD(P)-binding Rossmann-fold superfamily protein	-1.6132	2.64E-68	1Avs2A	Down
GSVIVT01022645001	Homeodomain-like superfamily protein	-1.2441	0.000004	1Avs2A	Down
GSVIVT01001207001	chlorophyllase 1	-1.5240	8.99E-11	1Avs2A	Down
GSVIVT01022645001	Homeodomain-like superfamily protein	-1.2441	4.83E-08	1Avs2A	Down
GSVIVT01030293001	ferritin 4	3.8684	1.65E-30	1Avs 3A	Up
GSVIVT01030294001	ferritin 2	2.4137	5.11E-07	1Avs 3A	Up
GSVIVT01025521001	Auxin-responsive family protein	-1.7239	1.12E-35	1Avs 3A	Down
GSVIVT01022645001	EXORDIUM like 2	-2.24869	1.68E-17	1Avs 3A	Down
GSVIVT01001207001	Ankyrin repeat family protein	-1.71366	2.54E-13	1Avs 3A	Down
GSVIVT01001205001	WRKY DNA-binding protein 40	-1.2013	3.55E-77	1Avs 3A	Down
GSVIVT01022645001	EXORDIUM like 2	-2.2487	1.68E-17	1Avs 3A	Down
GSVIVT01032754001	Serine/threonine-protein kinase WNK (With No Lysine)-related	1.12296	0	1Avs 3A	Up
GSVIVT01030293001	ferritin 4	3.70123	4.63E-28	1Avs 4A	Up
GSVIVT01030291001	ferritin 4	3.31173	4.33E-31	1Avs 4A	Up
GSVIVT01030294001	ferritin 2	1.76834	0.000727	1Avs 4A	
GSVIVT01007545001	Radical SAM superfamily protein	1.64335	8.86E-75	1Avs 4A	Up
GSVIVT01028900001	NAD(P)-binding Rossmann-fold superfamily protein	-1.36480	1.67E-12	1Avs 4A	Down
GSVIVT01007996001	Pyridine nucleotide-disulphide oxidoreductase family protein	1.00873	0	1Avs 4A	Up
GSVIVT01037438001	Pyridine nucleotide-disulphide oxidoreductase family protein	-1.31470	2.20E-07	1Avs 4A	Down
GSVIVT01007996001	Pyridine nucleotide-disulphide oxidoreductase family protein	1.00873	0	1Avs 4A	Up
GSVIVT01037438001	Pyridine nucleotide-disulphide oxidoreductase family protein	-1.31470	2.20E-07	1Avs 4A	Down
GSVIVT01008862001	Pheophorbide a oxygenase family protein with Rieske [2Fe-2S] domain	-1.0382	1.05E-12	1Avs 4A	Down
GSVIVT01001374001	myb-like HTH transcriptional regulator family protein	-3.52758	2.26E-16	1Avs 4A	Down
GSVIVT01001207001	chlorophyllase 1	-1.96639	1.52E-17	1Avs 4A	Down
GSVIVT01032652001	methyl esterase 17	-1.7304	2.74E-84	1Avs 4A	
GSVIVT01024667001	ferritin 2	-2.61555	0.000292	2Avs 3A	Down
GSVIVT01025521001	NAD(P)-binding Rossmann-fold superfamily protein	-1.20585	1.83E-14	2Avs 3A	Down
GSVIVT01007545001	Radical SAM superfamily protein	1.37563	6.57E-55	2Avs 4A	Up
GSVIVT01019612001	NAD(P)-binding Rossmann-fold superfamily protein	1.97464	4.89E-11	2Avs 4A	
GSVIVT01028900001	NAD(P)-binding Rossmann-fold superfamily protein	-1.97586	0	2Avs 4A	Down
GSVIVT01037438001	Pyridine nucleotide-disulphide oxidoreductase family protein	-1.69274	1.69E-12	2Avs 4A	Down
GSVIVT01010106001	NYC1-like	1.28498	1.04E-10	2Avs 4A	Up
GSVIVT01001374001	myb-like HTH transcriptional regulator family protein	-3.20441	5.98E-12	2Avs 4A	Down
GSVIVT01032652001	methyl esterase 17	-1.4846	3.64E-55	2Avs 4A	Down
GSVIVT01010106001	NYC1-like	1.28498	1.04E-10	2Avs 4A	Down
GSVIVT01015900001	Homeodomain-like superfamily protein	-1.46670	1.79E-11	2Avs 4A	Down
GSVIVT01030294001	ferritin 2	-1.32534	1.30E-05	3Avs 4A	Down
GSVIVT01019612001	NAD(P)-binding Rossmann-fold superfamily protein	2.08062	7.16E-12	3Avs 4A	Up
GSVIVT01025521001	NAD(P)-binding Rossmann-fold superfamily protein	1.22868	2.05E-16	3Avs 4A	Up
GSVIVT01028900001	NAD(P)-binding Rossmann-fold superfamily protein	-2.28338	0	3Avs 4A	Down
GSVIVT01037438001	Pyridine nucleotide-disulphide oxidoreductase family protein	-1.94636	1.37E-17	3Avs 4A	Down
GSVIVT01022645001	Homeodomain-like superfamily protein	1.31476	2.73E-05	3Avs 4A	Up
GSVIVT01010106001	NYC1-like	1.09996	1.10E-80	3Avs 4A	Up
GSVIVT01001374001	myb-like HTH transcriptional regulator family protein	-3.12037	4.53E-11	3Avs 4A	Down
GSVIVT01015900001	Homeodomain-like superfamily protein	-1.56509	2.47E-13	3Avs 4A	Down
GSVIVT01032652001	methyl esterase 17	-1.40082	3.75E-48	3Avs 4A	Down
GSVIVT01022645001	Homeodomain-like superfamily protein	1.31476	2.73E-05	3Avs 4A	Up
GSVIVT01010106001	NYC1-like	1.09996	1.10E-80	3Avs 4A	Up
GSVIVT01001374001	myb-like HTH transcriptional regulator family protein	-3.12037	4.53E-11	3Avs 4A	Down
GSVIVT01015900001	Homeodomain-like superfamily protein	-1.56509	2.47E-13	3Avs 4A	Down

Transcripts with in all developmental stages, 25 showed changed expression levels form 1.01 (GSVIVT01020661001 |log2FC| = 1.01) to 4.68 (GSVIVT01000112001, |log2FC| = 4.68) during stage 2A. The expression levels in 1Avs2A, GSVIVT01028043001 (117.6172085) GSVIVT01038557001 (90.85667248), GSVIVT01008673001 (82.22664817), and 3A vs 4A GSVIVT01008398001 (93.25810357), GSVIVT01028152001 (87.32409097) were observed. These were enriched during the initial leaf growth, which integrated genes that were highly up-regulated in immature leaves compared towards maturity, and is in harmony with the preliminary findings that number of genes involved in photosynthetic pathway were specifically expressed throughout developmental phase. The results indicated that with the leaf growth reduced chlorophyll content because of the chlorophyll synthesis rate is reduced at the later stages as compared to early leaf growth, which also shows that the chlorophyll signaling genes expression is lower.

In the grapevine transcriptome, 24 photosynthesis-related transcripts were identified during leaf growth stages involving in photosystem I reaction center subunit (5), photosystem II reaction center protein (12), CemA-like proton extrusion protein-related (2), photosynthetic electron transfer C (3), electron carriers (1) and Cytochrome c (1). Among the 5 differentially expressed genes of photosystem I, PsbY, PsaG, PsaN, PasO were up-regulated and PSAN was down-regulated (Table 4). During the intentional developmental stages photosystem I showed down regulated expression, while at lateral stages significantly increased. 12 differentially expressed transcripts of photosystem II reaction center proteins almost all showed upregulation as compared to initial leaf growth stages, and Cem A-like proton extrusion protein-related showed significantly up-regulated. Meanwhile photosynthetic electron transfer C was down-regulated in all developmental stages “S2 File”. Electron carriers and Cytochrome c were significantly up-regulated. We validated the developmental stages of flower, fruit and leaf and observed differentially expressed transcripts by using qRT-PCR. The results were about constant with the expression of photosynthesis and chlorophyll metabolic pathway “S3 File”.

**Table 4:** Differential expressed genes, related to photosynthesis metabolic pathway.

Gene ID	Trait	Log2FC	FDR	Leaf stage	Gene annotation	Result
GSVIVT01026478001	photosystem I reaction center subunit PSI-N, chloroplast, putative / PSI-N, putative (PSAN)	-1.00324	0	1Avs2A	PSAN	Down
GSVIVT01006662001	photosystem II reaction center protein C	0.66194	0.0821	1Avs3A and 4A	PsbC	up
GSVIVT01022171001	photosystem II reaction center protein C	-0.70627	9.70E-14	1Avs3A	epsilon	Down
GSVIVT01031113001	Photosystem II reaction center PsbP family protein	-0.12803	0.1033	1Avs 4A	PsbP	up
GSVIVT01029875001	CemA-like proton extrusion protein-related	1.58193	0.00002	1Avs 4A	PetA	Up
GSVIVT01021650001	root FNR 2	1.02786	0	1Avs 4A	PetH	Up
GSVIVT01006662001	photosystem II reaction center protein C	1.44747	6.36E-07	1Avs 4A	PsbC	up
GSVIVT01015713001	photosynthetic electron transfer C	-1.74158	0	1Avs 4A	PetC	Down
GSVIVT01029661001	photosystem II reaction center protein D	1.97122	0.00002	2A vs 4A	PsbD	Up
GSVIVT01009624001	photosystem II subunit O-2	1.32834	0	2A vs 4A	PsbO	Up
GSVIVT01031268001	Photosystem II reaction center PsbP family protein	1.20752	2.88E-79	2A vs 4A	PsbP	Up
GSVIVT01031268001	Photosystem II reaction center PsbP family protein	1.20752	2.88E-79	2A vs 4A	PsbW	Up
GSVIVT01029661001	photosystem II reaction center protein D	1.97122	2.43E-05	2A vs 4A	PsbY	Up
GSVIVT01001159001	photosystem I subunit D-2	1.57268	0	2A vs 4A	PsaD	Up
GSVIVT01014842001	photosystem I subunit G	1.0039	0	2A vs 4A	PsaG	Up
GSVIVT01026478001	photosystem I reaction center subunit PSI-N, chloroplast, putative / PSI-N, putative (PSAN)	1.1643	0	2A vs 4A	PsaN	Up
GSVIVT01036077001	photosystem I subunit O	1.02812	0	2A vs 4A	PasO	Up
GSVIVT01029875001	CemA-like proton extrusion protein-related	1.7206	1.41E-05	2A vs 4A	PetA	Up
GSVIVT01012423001				2A vs 4A	PetB	Down
GSVIVT01015713001	photosynthetic electron transfer C	-1.2588	0	A2 vs 4A	PetC	Down
GSVIVT01000982001				2A vs 4A	PetF	Down
GSVIVT01016441001	photosystem II reaction center protein A	1.2438	4.92E-78	3A vs 4A	PsbA	Up
GSVIVT01006503001	photosystem II reaction center protein B	1.2338	4.91E-11	3A vs 4A	PsbB	Up
GSVIVT01006217001	electron carriers	1.25475	0.00025	3A vs 4A	PsbE	Up
GSVIVT01001159001				3A vs 4A	PsaD	Up
GSVIVT01012076001	Cytochrome c	1.02045	5.04E-17	3A vs 4A	PetJ	Up
GSVIVT01015713001	photosynthetic electron transfer C	-1.42937	0	3A vs 4A	PetC	Down

### Genes related to secondary metabolism biosynthetic pathways

Syntheses of secondary metabolites in leaves are important part of the growth and development of plants. An analysis of leaf developmental stages specify that secondary metabolites and their pathways generally were highly up-regulated in early stages of growth, as the biosynthesis mechanism of metabolites were increased. In the present experiment, 489 secondary metabolites related genes were identified during leaf growth, which includes alkaloid (40), anthocyanins (21), Diterpenoid (144), Monoterpenoid (90) and Flavonoids (93).

Alkaloid and alkaloid compounds were significantly expressed during leaf development. In alkaloid biosynthesis pathway, 24 and 18 transcripts were up and down-regulated respectively, which includes Calcium-dependent phosphotriesterase superfamily protein having 5 up-regulated and 2 down-regulated transcripts, Tyrosine transaminase family protein having 4 up-regulated, methyl esterase 10 showed 6 up-regulated transcripts and NAD(P)-binding Rossmann-fold superfamily protein having 5 up-regulated and single transcript was down-regulated “S3 File”.

Anthocyanins, acting as powerful antioxidants, are class of flavonoid compounds, which scavenge diverse reactive oxygen species or inhibit their formation by chelating pro-oxidative metal ions [23]. In anthocyanins biosynthetic pathways, 13 and 19 transcripts, respectively up-regulated and down-regulated in respond to leaf development and accumulation of anthocyanin. During the early stages of growth, generally transcripts were up-regulated and on lateral stages they were down regulated, so it shows that the production and accumulation of anthocyanin regulating genes were highly active and most probably the biosynthesis was complete in the early stages as compared to maturity. Anthocyanins biosynthetic transcripts mainly including UDP-Glycosyl transferase super family protein having 6 up-regulated and 8 down-regulated transcripts “S3 File”.

Terpenoids play key role in pollinator attraction, plant resistance, and interaction with the surrounding environment. Cytosolic mevalonic-acid (MVA) and plastidial 2-C-methylerythritol 4-phosphate (MEP) pathway are responsible for the biosynthesis of these compounds [24]. In our study, diterpenoid biosynthesis pathway 53 and 83 genes were significantly up-regulated and down-regulated respectively, including 2-oxoglutarate (2OG) and Fe(II)-dependent oxygenase superfamily protein with 13 and 17 up-regulated and down-regulated transcripts respectively, cytochrome P450, family having 7 up-regulated and 18 down-regulated transcripts, terpene synthase 21 having 2 up-regulated and 5 transcripts were down-regulated on lateral stages and Terpenoid cyclases/Protein prenyltransferases superfamily protein having 6 down-regulated transcripts only. We validated the transcripts expression of differently expressed transcripts using qRT-PCR at varying growth stages of leaves, flower and fruits. The results predicted that transcripts are specific to the growth stage and have almost same expression as we observed in secondary metabolic pathway for Terpenoids, Anthocyanins, Alkaloid and alkaloid compounds.

Recently, efforts have been made to metabolically engineer monoterpene biosynthesis. Especially in Arabidopsis [25] and Tobacco [26, 27], monoterpenes were produced through the action of terpene synthase-a (TPS-a; [28] enzymes that generally use pyrophosphate as a substrate, develops from products of the deoxy xylulose-5-phosphate (DXP) pathway, dimethylallyl pyrophosphate (DMAPP) and isopentenyl pyrophosphate (IPP). The DXP synthesis pathway consist of seven different chloroplast-localised enzymes [29], for which 6 of them encoding transcripts were expressed with little differential regulation at all four developmental stages [16]. In the present study Monoterpenoid showed 18 up-regulated and 83 down-regulated transcripts, Monoterpenoid including, NAD (P)-binding Rossmann-fold superfamily protein with 4 up-regulated 18 down-regulated transcripts, terpene synthase 03 having 5 up-regulated and 17 down-regulated, terpene synthase showed 14 up-regulated and 25 down-regulated transcripts and terpene synthase-like sequence-1, 8-cineole having 2 and 7 were up and down regulated respectively (S3 File). Flavonoids based on the relatively 87 transcripts were up-regulated and 107 down-regulated transcripts, including cytochrome P450, family 82, polypeptide 4, subfamily C, having 15 up-regulated and 12 down-regulated, HXXXD-type acyl-transferase family protein 9 up-regulated and 18 down-regulated, senescence-related gene 1, 2 up-regulated and 7 down-regulated, 2-oxoglutarate (2OG) and Fe(II)-dependent oxygenase superfamily protein having 2 and 24 up and down-regulated transcripts were recorded respectively “S3 File”.

### Genes related to Plant hormone signal transduction pathways during leaf developmental stages

The most enrichment and significantly expressed transcripts involved in expression of hormonal signaling, which were highly enriched in 1Avs2A and 1Avs3A growth stages. This might be suggesting that generally, hormone function and signaling is controlled by metabolic networks are mostly to be activated during the initial growth stages. Moreover, since terms concerning to ribosome biogenesis, translational elongation and nucleosome assembly are enriched 72 and 118 transcripts were significantly up-regulated and down-regulated, respectively (Table 5), it was observed that additional translationally active through premature growth than afterward in the developmental phase. This could be due to the cell division with high rate of differentiation, which later reduced as growth increasingly come as regards through cell expansion and vacuolar expansion [30]. We compared these transcripts profiles with those associated with other signaling molecules and phytohormones, namely SAUR-like auxin-responsive protein family having 2 up-regulated and 7 down-regulated, phytochrome-associated protein showed 1 up-regulated and 9 transcripts were down-regulated, Auxin-responsive GH3 family protein 1 up-regulated and 4 transcripts were down-regulated, while genes associated with the Leucine-rich repeat protein kinase family protein showed 7 up-regulated and 1 down-regulated transcript, like Auxin Resistant 2, only one showed up regulation in second developmental stage and 3 were down-regulated at lateral stages (3A and 4A), UX/IAA transcriptional regulator family protein expressed 4 up-regulated and 7 down-regulated transcripts, SAUR-like auxin-responsive protein family have 5 up and 6 down regulated, myb-like HTH transcriptional regulator family protein showed 3 up-regulated and 2 down-regulated transcripts. We found that genes associated with the signaling molecules like Auxin Resistant, SAUR-like auxin-responsive protein family, UX/IAA transcriptional regulator family protein and Leucine-rich repeat protein kinase family protein were significantly down-regulated among the leaf samples and up-regulated during the growing stages (Table 5, S2 File). The expression pattern was confirmed by qRT-PCR and validated the observed transcripts at developmental stages of leaf, flower and fruit, differential expression of signal transduction transcripts using qRT-PCR (Fig 4). We confirmed that the transcript expression is extremely specific to the developmental stages of leaf; flower and fruit were consistent amongst the studied samples, which showed high expression during early stages and have lower expression at later stages in some transcripts.

**Table 5:** Information of differential expression genes related to Plant hormone signal transduction pathways at developmental stages.

Description	No. of up-regulated	No. of down-regulated	Sum
S-locus lectin protein kinase family protein	4	1	5
Leucine-rich repeat protein kinase family protein	7	1	8
cysteine-rich RLK (RECEPTOR-like protein kinase) 2	2	2	4
histidine-containing phosphotransfer factor 5	4	0	4
S-locus lectin protein kinase family protein	4	1	5
Concanavalin A-like lectin protein kinase family protein	2	1	3
indole-3-acetic acid inducible 14	0	3	3
AUX/IAA transcriptional regulator family protein	4	7	11
indole-3-acetic acid inducible 19	0	2	2
O-Glycosyl hydrolases family 17 protein	1	7	8
alpha/beta-Hydrolases superfamily protein	3	6	8
carboxyesterase 17	3	2	5
carboxyesterase 20	4	1	5
myb-like HTH transcriptional regulator family protein	3	2	5
Homeodomain-like superfamily protein	1	2	3
response regulator 11	1	1	2
Transcriptional factor B3 family protein / auxin-responsive factor AUX/IAA-related	1	0	1
Protein kinase superfamily protein	1	3	4
like AUXIN RESISTANT 2	1	2	3
Transmembrane amino acid transporter family protein	0	5	5
phytochrome-associated protein 1	0	5	5
phytochrome-associated protein 2	1	4	5
indole-3-acetic acid inducible 29	0	2	2
histidine-containing phosphotransfer factor 5	4	0	4
histidine-containing phosphotransmitter 1	4	0	4
Carbohydrate-binding X8 domain superfamily protein	0	5	5
highly ABA-induced PP2C gene 3	1	0	1
Protein phosphatase 2C family protein	1	7	8
Auxin-responsive GH3 family protein	1	3	4
CAP (Cysteine-rich secretory proteins, Antigen 5, and Pathogenesis-related 1 protein) superfamily protein	1	0	1
xyloglucan endotransglycosylase 6	0	6	6
xyloglucan endotransglucosylase/hydrolase 16	0	3	3
xyloglucan endotransglucosylase/hydrolase 24	0	2	2
indoleacetic acid-induced protein 16	0	2	2
indole-3-acetic acid 7	0	3	3
indole-3-acetic acid inducible 30	0	2	2
HPT phosphotransmitter 4	3	0	3
SAUR-like auxin-responsive protein family	11	0	11
carboxyesterase 18	1	2	3
TIP41-like family protein	2	0	2

## Discussion

Next to genetic insight, increased computing clout, for instance through improved parallelization algorithms, will likely become the limiting factor in the developmental process. Eventually, a mechanistic model for leaf growth and development should assimilate the regulatory networks that direct developmental processes and decisions of cells as they migrate in space and time from the shoot apical meristem to their final position in the leaf growth [6]. Through photosynthetic activities of the leaf provides the basis for growth and development during the entire life of plants. The development of plant leaf is dynamic mechanism, where self-determining regulatory pathways instruct component of cells at diverse developmental stages to make differentiation switches and to control the rate at which growth process is completed. When integrated over the intact cell population in the leaf, cell enlargement and ultimately size and shape are developing properties that can be evaluated to real leaves growth [6, 10]. It has been hypothesized that there are number of networks that regulates various environmental and autonomous factors, and these pathways are probably interrelated and integrated to form a regulatory networks to control leaf growth, development and senescence [31]. During initial growth (1A and 2A) phases the transcripts were significantly up-regulated, while at the next growth stages, photosynthetic activities were significantly decreased in the leaves of 3A and 4A as compared to 1A and 2A grapevine leaves. Although, PsbB, PsbE and PsaD showed upregulation during lateral growth phases, meanwhile, PetF, PetC and PetB were down-regulated compared to 1A and 2A in photosynthetic pathway. The values of photosystem II and PSI-N reflects the potential quantum efficiency and are frequently used as a responsive indicator of photosynthetic performance as same in higher plant leaves [32]. The result were consistent with previous reports that PSI was usually more stable than PSII [33]. Photosynthetic activities and chlorophyll pigmentation are greatly reduced with the days of leaves growth. In order to validate this presumption in grapevine, we investigated the photosynthetic and chlorophyll content in current study, which showed a significant decrease in chlorophyll content and photosynthetic activities from in transcriptomic level and physiological processes. Furthermore, transcriptomic data demonstrated that the leaf growth stages have significant difference and chlorophyll biosynthesis enzymatic activity of chlorophyll degradation enzymes. The decline in chlorophyll content is believed to be due to acceleration in chlorophyll II with leaf maturity and degradation of cells [34] and/or the blocking of chlorophyll synthesis [35]. This statement also supported by the finds of Fasoil et al., [5] that he pollen transcriptome was highly distinctive as was the transcriptome of the leaf undergoing senescence. During Chlorophyll metabolism and production pathway, Serine/threonine-protein kinase WNK Radical, shoot apical meristem (SAM) superfamily protein were up regulated while only two chlorophyllase I and 5 NAD(P) were significantly differentially-expressed during the initial stages, while later showed up regulation with the leaf growth. It shows that the expression of genes at the first phase of development had no specific function although with the progress in growth, genes were highly expressed. Photosystem stoichiometry regulation is a long-term feedback that involves hours to date. Transmits the excitation difference by altering the relative amounts in both photosystems [36]. The mRNA and protein accumulation were decreasing corresponded directly. Due to LSU and SSU proteins reduced speedily following, the disappearance of their corresponding transcripts, this would specify that these proteins were turned over comparatively rapidly in the chloroplasts of mesophyll cells. Although many researchers reported that the RuBPCase proteins are extremely stable with half-lives on the order of days [37, 38]. It might be promising that the constancy of these proteins in the two cell types is differentially distorted in response to developmental signals. RuBPCase may become selectively turned over in mesophyll cells or selectively stabilized in bundle sheath cells so that different amounts accumulate in the two cell types as the leaves expand and developed [37].

Flavonoids are omnipresent plant secondary metabolites that including flavonols, flavones, anthocyanins, flavandiols, condensed chalcones, tannins (or proanthocyanidins) and aurones. Flavonoids having considerable UV absorption abilities and reduction of UV radiation is the result of amalgamation and distribution within epidermal tissue [39]. In addition, it was reported that involved in many unambiguous biological processes, such as signaling, that have strict substrate specificity as well as in other different physiological activities [40, 41]. The shikimate pathway, a link central metabolism and secondary metabolism, is the only reported biosynthetic pathway for synthesis of aromatic amino acids (Try, Phe and Tyr) and the chorismate, which serve as key precursor for a wide range of secondary metabolites, such as alkaloid, phenolic and plant hormones [42, 43]. The major group of these secondary metabolites is the phenylpropanoids, whose biosynthesis is initiated by the activity of Phenylalanine ammonia lyase (PAL). PAL is the first and committed enzyme in the phenylpropanoid biosynthesis pathway; therefore, a key step in the biosynthesis of the favonoids, lignins, stilbenes and many other compounds [44]. In the present study, anthocyanins biosynthetic pathways, 13 transcripts were significantly up-regulated and 19 were significantly down-regulated. Most related genes in the anthocyanins biosynthesis, UDP-glucosyl transferase 78D2, indole-3-acetate were also significantly up-regulated in 1Avs2A and 3A,while in the 3Avs4A showed significantly down-regulated, illustrated secondary metabolites that are often associated with in respond to leaf development and accumulation of anthocyanin. Although Monoterpenoid 18 genes were significantly up-regulated and 83 were significantly down-regulated. In case of Flavonoids 87 transcripts were up-regulated and 107 were significantly down-regulated, as well as cytochrome P450, family 82, subfamily C, polypeptide 4, having 15 up-regulated and 12 down-regulated. In our study Diterpenoid biosynthesis pathway 53 and 83 genes were up-regulated and down-regulated respectively, including 2-oxoglutarate (2OG) and Fe(II)-dependent oxygenase superfamily protein with 13 up-regulated and 17 were down-regulated, depicting that with the leaf development and cell multiplication the rate to secondary metabolites activities and synthesis gradually decrease. The research findings suggested that production of secondary metabolites in regent may be more trigger than in Trincadeira. Furthermore, CAD down-regulation in regent proposed that phenolic composites were not polymerized into lignin; therefore, the common phenylpropanoid biosynthetic pathway leading to the growth of phenolic compounds might be more active than that of the monolignol-specific pathway [45].

Phytohormones, such as auxin, cytokines and abscisic acid (ABA) have critical role in plant leaf growth and development. Several mathematical modeling findings [46], have replicated phyllotactic prototype based on response to exchanges among auxin and PIN allocation. A number of models hypothesize that AUX1creates auxin accumulation mostly in L1laye cells, where PIN1 is primarily restricted in the protodermal (L1) layer cells and causes drainage of auxin to the bases of the shoots by inducing vascular strand segregation in L2/3layer cells of the shoot apical meristem [47]. In the recent work, 72 genes were significantly up-regulated and 118 were significantly down-regulated in hormone signaling pathway, namely SAUR-like auxin-responsive protein family having 2 up-regulated and 7 down-regulated, SAUR-like auxin-responsive protein family have 5 up and 6 down-regulated, UX/IAA transcriptional regulator family protein expressed 4 up-regulated and 7 down-regulated transcripts, myb-like HTH transcriptional regulator family protein showed 3 up-regulated and 2 down-regulated transcripts. In addition Cytokinins control cell division and a variety of metabolic and growth processes, including senescence. Abscisic acid (ABA) and cytokinin are key hormones controlling plant development. We found that genes associated with the signaling molecules like Auxin Resistant, SAUR-like auxin-responsive protein family, UX/IAA transcriptional regulator family protein and Leucine-rich repeat protein kinase family protein were significantly down-regulated among the leaf samples and up-regulated during the growing stages. AUX/IAA proteins and auxin responsive factors (ARFs) regulated genes related to the transcription of auxin-induced [48]. Our findings are more appropriate with Brenner, Romanov [49], in *Arabidopsis* model plant, where 71 up-regulated and 11 down-regulated immediate CK-response genes were reported. Initially, progenitor cells are outside the stem cell niche, they have to choose whether they will participate to the main axis or will differentiate in to lateral appendices such as leaf primordia. This decisions primarily governed by the accumulation of the plant hormone auxin its PIN-FORMED1 (PIN1) efflux transporter and its influx carrier AUXINRESISTANT (AUX1) [50]. Certainly, mutations in CK receptors and overexpression of the CK dehydrogenase gene family of Arabidopsis (AtCKX) reduce leaf area and meristem size [51], indicating a relation among the leaf size and shoot apical meristem. It appears however that the number of leaf originator cells is not an essential determinant of the ultimate leaf size.

## Conclusion

Results of present study have better defined the biosynthetic pathways of chlorophyll, photosynthesis, hormonal signaling and secondary metabolites specifically by identifying the majority of the transcripts that are involved in the biological and biochemical process and hormone signaling. The identified transcripts in this work and their functional characterization at molecular, cellular and biochemical level will be helpful to understand the biosynthetic pathway and its regulation at growth and developmental stages in higher plants. Through the use of illumine sequencing; we observed the detail transcriptional changes that occur during grape leaf development and validated the differentially expressed transcripts via qrtPCR technique.

## Supporting Information

S1 File Primers used for real time PCR analysis. (XLSX)

S2 File Differentially expressed genes in all developmental stages (cellular component, molecular function and biological process). (XLSX)

S3 File KEGG Biosynthetic Pathways (Chlorophyll metabolism pathway, Plant Horomone signal Transduction and Photosynthesis Biosynthetic Pathways) and Secondary metabolic biosynthetic pathways (Indole Alkaloid Biosynthetic Pathway, Isoquinoline alkaloid biosynthesis, Anthocyanins Biosynthetic Pathway, Diterpenoid Biosynthesis, Monoterpenoid Biosynthesis, Flavonoids Biosynthesis). (XLS)
